# Authors’ Reply to: Strengthening the Backbone: Government-Academic Data Collaborations for Crisis Response

**DOI:** 10.2196/66479

**Published:** 2024-11-28

**Authors:** Jian-Sin Lee, Allison R B Tyler, Tiffany Christine Veinot, Elizabeth Yakel

**Affiliations:** 1 School of Information, University of Michigan, Ann Arbor, MI, United States; 2 UK Data Archive, University of Essex, Colchester, United Kingdom; 3 Department of Health Behavior and Health Equity, School of Public Health, University of Michigan, Ann Arbor, MI, United States; 4 Department of Learning Health Sciences, School of Medicine, University of Michigan, Ann Arbor, MI, United States

**Keywords:** COVID-19, crisis response, cross-sector collaboration, data infrastructures, data science, data sharing, pandemic, public health informatics

In their *Letter to the Editor* responding to our original viewpoint article [1], Yang and Yang [2] identified practical challenges and limitations inherent in constructing the government-academic data infrastructures that we proposed. They emphasized the importance of further examining the nuances involved in implementing this vision. We appreciate their thoughtful feedback and are pleased that our work has sparked the kind of dialogue that we intended. In this *Authors’ Reply*, we engage with the issues raised in the letter while offering extended discussions based on these comments.

Indeed, logistical and ethical challenges, such as privacy concerns when sharing sensitive data during crises, have long been central to ongoing debates. We agree that comprehensive data governance policies will be a critical cornerstone for enabling government-academic collaboration. To this end, we urge leaders of such partnerships to carefully balance public health research imperatives and individual consent, as well as, importantly, group privacy [3], when developing data policies. This delicate balance could be achieved by drawing on frameworks such as the CARE Principles for collective rights [4] or data protection principles in humanitarian contexts.

The authors’ considerations regarding sustained funding and long-term human resource planning are pragmatic. We acknowledge that underinvestment in public health infrastructures poses a major limitation to government-academic data collaborations. Nonetheless, the US Centers for Disease Control and Prevention’s (CDC) recent establishment of the Outbreak Analytics and Disease Modeling Network (OADMN) [5] represents a promising attempt to foster such partnerships at the state level. The question remains: How can these efforts be made sustainable? One critical step is to demonstrate their ongoing value. Additionally, it is crucial to heed the lessons from the early 2010s, when the CDC’s public health informatics program, noted in our original article, was defunded.

Addressing the barriers related to collecting, processing, and analyzing public health data constitutes a fundamental rationale for our advocacy of more “direct,” project-based government-academic collaboration. Our perspective aligns closely with the author’s recognition of the pivotal role of reciprocal, bidirectional learning and capacity building within such partnerships. Whether in formulating cross-sector data standardization protocols or ensuring that data-derived results effectively inform public health measures, collaborating entities must communicate closely about local needs and case-specific situations, mutually contributing their expertise and resources. A bottom-up approach, informed by a deeper understanding of similar collaborations, could provide valuable insights for the data science training programs already in place within public health departments and health informatics education settings.

Realizing the vision of robust and sustainable government-academic data infrastructures requires overcoming practical challenges, and it cannot be accomplished overnight. With this *Authors’ Reply*, we again call for more practice-based, action-oriented case studies on the types of partnerships in question. As one such attempt, the findings from our empirical investigation of a state-level case study will soon be shared with the scientific community, further illuminating the intricate dynamics at play.

## Abbreviations

CDC: US Centers for Disease Control and Prevention
OADMN: Outbreak Analytics and Disease Modeling Network
